# Autochthonous chikungunya virus (CHIKV) outbreak in the province of Modena, Emilia-Romagna region, Italy, August to October 2025: epidemiology, clinical features and virological findings

**DOI:** 10.2807/1560-7917.ES.2026.31.36.2500962

**Published:** 2026-09-10

**Authors:** Giada Rossini, Giulio Matteo, Paola Angelini, Mattia Calzolari, Rodolfo Veronesi, Alessandra Fantuzzi, Caterina Vocale, Silvia Galli, Liliana Gabrielli, Michele Dottori, Riccardo Mazzoli, Tommaso Filippini, Maurilia Marcacci, Guido Di Donato, Giovanna Mattei, Tiziana Lazzarotto, on behalf of the Emilia-Romagna CHIKV outbreak group

**Affiliations:** 1 Microbiology Unit, IRCCS Azienda Ospedaliero-Universitaria di Bologna, Bologna, Italy; 2 Emilia-Romagna Region Public Health Department, Bologna, Italy; 3 Istituto Zooprofilattico Sperimentale della Lombardia e dell'Emilia Romagna “B. Ubertini”, Brescia, Italy; 4 Medical and Veterinary Entomology Department, Centro Agricoltura Ambiente “G. Nicoli”, Crevalcore, Italy; 5 Department of Public Health - Public Hygiene Service, Local Health Authority of Modena, Modena, Italy; 6 Department of Biomedical, Metabolic and Neural Sciences, University of Modena and Reggio Emilia, Modena, Italy; 7 School of Public Health, University of California Berkeley, Berkeley, the United States; 8 Istituto Zooprofilattico Sperimentale dell’Abruzzo e del Molise, Teramo, Italy; 9 Department of Medical and Surgical Sciences (DIMEC), University of Bologna, Bologna, Italy; 10 The members of the Emilia-Romagna CHIKV outbreak group are listed under Acknowledgements

**Keywords:** arthropod-borne virus, autochthonous, Chikungunya virus

## Abstract

Between 10 August and 31 October 2025, 343 cases (306 confirmed and 37 probable) of symptomatic autochthonous chikungunya virus (CHIKV) infection were notified in eight municipalities in the province of Modena, Emilia-Romagna region, northern Italy. The infection was diagnosed by detection of CHIKV RNA in blood and urine samples, and by detection of IgM antibodies against CHIKV. Most common symptoms were arthralgia (n = 326) and fever (n = 317). No fatalities were reported. Chikungunya virus RNA was detected in 39 (14.8%) of 263 pools of *Aedes albopictus* mosquitoes, thereby confirming local vector-borne transmission. Sequences from 11 case samples and two mosquito pools were whole genome sequenced. The viral strain belonged to the East/Central/South African (ECSA) lineage 2 and was closely related to the strains circulating in Reunion Island in 2024–25. The public health response included rapid vector control measures, reinforced epidemiological surveillance and multidisciplinary coordination between public health authorities and clinicians, laboratories and entomologists. This was one of the largest autochthonous CHIKV outbreaks ever recorded in a temperate European region. It highlights the critical importance of integrated, multidisciplinary preparedness and response for arboviral threats in non-endemic areas.

**Key public health message**

**What did you want to address in this study and why?**

Chikungunya is a viral disease transmitted via mosquitoes and presenting with fever and severe joint pain. The virus is usually found in Asia, Africa and the Americas. We wanted to describe a large outbreak of chikungunya which occurred in the Modena province of northern Italy between August and October 2025, affecting at least 343 people across eight municipalities.

**What have we learnt from this study?**

This was one of the largest ever recorded chikungunya outbreaks in mainland Europe. Almost all identified cases presented with joint pain and fever. Chikungunya virus was detected also in mosquitoes, thereby confirming ongoing local transmission. The virus from this outbreak was closely related to those from Reunion Island, thus suggesting the virus was imported via a traveller.

**What are the implications of your findings for public health?**

European regions, where the vector Asian tiger mosquito is established, are at an increasing risk of chikungunya virus and other arbovirus outbreaks, particularly as viral circulation expands in endemic areas. An effective preparedness requires a combination of heightened awareness among healthcare professionals and laboratories for early detection of patients and timely implementation of outbreak control measures.

## Background

Chikungunya virus (CHIKV) is a single-stranded, positive-strand RNA arbovirus of the *Alphavirus* genus within the *Togaviridae* family [1]. Infection with CHIKV presents a broad clinical spectrum, from asymptomatic infection to severe and potentially life-threatening disease. Symptomatic cases typically manifest with acute-onset high fever, non-pruritic maculopapular rash and severe polyarthralgia. In some patients, arthralgia persists for months or years, resulting in considerable chronic morbidity. The virus is transmitted to humans through the bites of infected *Aedes* spp. mosquitoes. In Europe, *Ae. albopictus* is established in the southern and central European countries and is thus the most relevant vector species.

Three main CHIKV genotypes have been identified: Asian, West African and East/Central/South African (ECSA) [1]. Chikungunya virus is endemic in Africa, South-eastern Asia, the Indian subcontinent, the Pacific region and tropical regions of the Americas [2]. In non-endemic regions, the introduction of the virus by viraemic travellers returning from areas of active transmission is a critical determinant of the outbreak risk. The probability of such introductions increases substantially during periods of high CHIKV circulation in endemic regions, highlighting the link between global CHIKV dynamics and outbreak risk in Europe. Outbreaks in Europe have all been caused by strains belonging to the ECSA genotype [2-7].

Although mainland Europe is not endemic for CHIKV, local transmission events have recurred since 2007 [2]. In Italy, *Ae. albopictus* has been firmly established for decades, and the ecological conditions required for local CHIKV transmission have been present since at least 2007. Italy is one of the European countries most severely affected, with the first large, documented outbreak in the Emilia-Romagna region in 2007 [3,4] and the second one in the Lazio region in 2017 [5,6]. In 2025, Reunion Island, a French Outermost Region in the Indian Ocean, experienced its largest epidemic in nearly two decades, with over 54,000 confirmed autochthonous cases [8], and France and Italy reported unprecedented clusters of autochthonous cases, consistent with the introductions of the virus by viraemic travellers arriving from the island [9]. France reported the highest numbers among the European countries, with more than 780 autochthonous cases and over 1,000 imported cases [9-12].

## Outbreak detection

On 8 August 2025, the Public Hygiene Service of Modena of the Emilia-Romagna region received notification from the infectious disease ward of Modena Polyclinic of a suspected case of chikungunya. The patient, a resident in Carpi (Modena province), in their forties, presented with fever, arthralgia, cutaneous rash, asthenia and myalgia, and with no recent travel history to endemic areas. On 10 August, samples from this person were sent to the Regional Reference Laboratory at the IRCCS Azienda Ospedaliero-Universitaria di Bologna which confirmed the presence of CHIKV RNA in the patient's serum, plasma and urine samples. Following the identification of this index case, all their close contacts (n = 4) also tested positive for CHIKV RNA in serum and/or plasma samples. Thus, the cluster threshold (≥ 2 linked confirmed cases) was reached. Following the confirmation of these cases, in line with the national (Piano Nazionale Arbovirus (PNA)) [13] and the Emilia-Romagna regional plan for surveillance and control of arboviruses (PSCA-ER) [14], the regional public health authorities set up a regional crisis unit comprising regional and local health authorities, the mayor, virologists, entomologists and other technical experts. The crisis unit met daily to implement and coordinate public health interventions. Here we describe the epidemiological, entomological and laboratory investigations and the public health response to the outbreak.

## Methods

### Case definition

Following the notification of the autochthonous cluster, a dedicated case definition was developed, based on national and regional plans for arboviruses [11,12] (Box), to promptly trace contacts and identify the affected areas.

Box **Definitions of suspected, probable and confirmed cases of infection with chikungunya virus, Emilia-Romagna, Italy, 2025**

**Suspected case:**

Clinical and epidemiological criteria:

• A person presenting with at least two of the following symptoms: fever > 38°C, arthralgia, skin rash, asthenia, retro-orbital pain, conjunctivitis, headache

AND

• Resident in Modena province and without travel history to an endemic country within 15 days before symptom onset

OR

• A person with an epidemiological link to a probable or confirmed case. Epidemiological link was defined as being a relative, living in the same household, school or day centre of a confirmed/probable case 15 days before and 7 days after symptom onset or a person regularly participating in group activities involving close physical contact to others for at least 5 consecutive hours per day.

**Probable case:**

Clinical and epidemiological criteria:

• As for a suspected case

AND

Laboratory criteria:

• IgM antibodies to CHIKV detected in a single serum sample.

**Confirmed case:**

Clinical and epidemiological criteria:

• As for a suspected case

AND

Laboratory criteria:

• CHIKV RNA detected in a sample

OR

• IgM antibodies to CHIKV detected in a single serum sample confirmed by neutralisation assay

OR

• Seroconversion

OR

• Fourfold increase in IgG antibodies in paired samples taken at least 2 weeks apart.

CHIKV: chikungunya virus.

### Chikungunya surveillance

In Italy, chikungunya is a notifiable disease, and surveillance is coordinated according to PNA [13] which provides guidelines for integrated entomological, epidemiological and laboratory monitoring, intensified during the vector season (May–October). The PSCA-ER [14] details local implementation including case management, laboratory workflow and coordination across public health networks. Chikungunya should be clinically suspected in patients presenting with acute-onset fever and severe arthralgia or arthritis not otherwise explained, particularly during the vector active season, irrespective of a documented history of travel to or residence in a CHIKV-endemic area. These cases are reported to local public health authorities and tested in regional reference laboratories. Confirmed cases trigger mandatory notification and vector control measures.

### Epidemiological investigation

All epidemiological investigations were conducted by the physicians and health assistants of the Public Health Service of Modena.

#### Case identification and field investigation

Cases were identified through active surveillance and notified to the regional SMI-PREMAL surveillance database. Suspected cases were reported by regional hospitals and general practitioners who were advised to suspect CHIKV infection in patients presenting with fever and/or arthralgia. Surveillance activities were promptly intensified across all regional healthcare settings. Residents eligible for door-to-door screening were identified using a geographical circular buffer with a 100 m radius around the residence of each confirmed case. All individuals residing within this area and presenting with symptoms consistent with the case definition, or reporting a recent relevant travel history, were eligible for screening.

#### Case interviews

All confirmed cases were interviewed by phone using a standardised trawling questionnaire. In the questionnaire, the questions had predominantly pre-defined response options (yes/no and multiple choice), with the possibility of adding free text and comments where appropriate. We collected following information and entered it into the regional SMI-PREMAL surveillance database: (i) demographic data (age, sex, place of residence including proximity to green spaces and apartment floor level); (ii) clinical details (type, onset and sequence of symptoms); (iii) exposure history (travel history, communal activities and potential high-risk contacts).

#### Contact tracing

Contacts identified via case interviews were subsequently interviewed and invited to undergo testing to detect secondary cases and asymptomatic infections. Samples from suspected cases were submitted to the Regional Reference Laboratory for analysis.

### Entomological investigations

Mosquitoes were collected with the human landing catch (HLC) method in which human volunteers collect mosquitoes that land on them. During each sampling session, mosquitoes were collected at 4–10 locations. Sampling was performed in accordance with the instructions from Centro Agricoltura Ambiente 'G. Nicoli' (https://www.caa.it/it/), ensuring personnel safety by exposing a small portion of one calf for 5 minutes per sampling location and using a battery-powered aspirator to collect the mosquitoes. Samples were collected (i) around the home of cases of new clusters (defined as ≥ 2 cases in which a spatio-temporal association was confirmed) before insecticidal treatment to assess the density of the vector population, as useful information to guide the decision on treatments (pre-treatment); (ii) as a control of effectiveness of insecticidal treatments (post-treatment). Following each intervention cycle, if consistently high vector densities were measured, treatments were replanned. The mean number of host-seeking mosquitoes collected per session was calculated and approximated to one unit (one mosquito). Mosquitoes from different sampling locations of one session were collected in the same aspiration tube and sent to the laboratory. Species identification of the collected mosquitoes was done morphologically [15].

### Virological and serological investigations

#### Patient samples

For detection of CHIKV RNA in serum, plasma and urine samples, we used the RealStar chikungunya RT-PCR Kit 2.0 AltoStar AM16 System (altona Diagnostics GmbH, Hamburg, Germany) and the cobas CHIKV test (Roche, Basel, Switzerland). Antibodies (IgM) against CHIKV in serum samples were detected using chikungunya VirClia Monotest (VirCell Microbiologists, Granada, Spain) and indirect immunofluorescence assay Anti-Chikungunya virus IFA (Euroimmun, Lübeck, Germany).

#### Mosquito samples

Prior to testing, adult mosquitoes were euthanised at −80°C for 5 minutes. Female and male mosquitoes (*Ae. albopictus*) were pooled separately (maximum 10 mosquitoes per pool). Thereafter, mosquito samples were mixed with 1 mL of PBS and two 4.5 mm round copper balls, vortexed and centrifuged at 4,000 g for 7 min. Nucleic acids extracted from 200 µL of supernatant were tested by a CHIKV real-time PCR assay [16]. Positive results were confirmed by the National Reference Centre for Foreign Animal Diseases (CESME). The maximum likelihood estimation of infection rate (IR) in collected samples was calculated by the PooledInfRate version 4.0 Excel add-in [17].

#### Sequencing and phylogenetic analysis

Samples from two mosquito pools and 11 sera with a quantification cycle (Cq) value < 30 were selected for whole genome sequencing (WGS). We used total RNA from the mosquito pools for sequencing with the SISPA protocol [18]. The libraries were prepared with the Illumina DNA Prep kit and sequenced on the NextSeq 1000 platform (Illumina, San Diego, the United States (US)). Mapping analysis was performed using the iVar tool version 1.4.4 [19] implemented in the GENPAT platform (https://genpat.izs.it/), with the reference sequence CHIKV Cameroon 2006 (NCBI accession number: KX262996.1). For serum samples, total RNA was extracted, reverse-transcribed and amplified using specific primers for targeted amplicon sequencing [20]. Libraries were generated using the Illumina Microbial Amplicon Prep Kit, then sequenced on the MiSeq platform (Illumina).

Complete genome reconstruction was performed using the Genome Detective application (https://www.genomedetective.com). Maximum likelihood (ML) phylogenetic reconstruction was performed using IQ-Tree (https://iqtree.github.io/) with the GTR model and 1,000 bootstrap replicates. The tree was rooted on the CHIKV ECSA Africa prototype.

## Results

### Epidemiological data and clinical features

**Figure 1:**
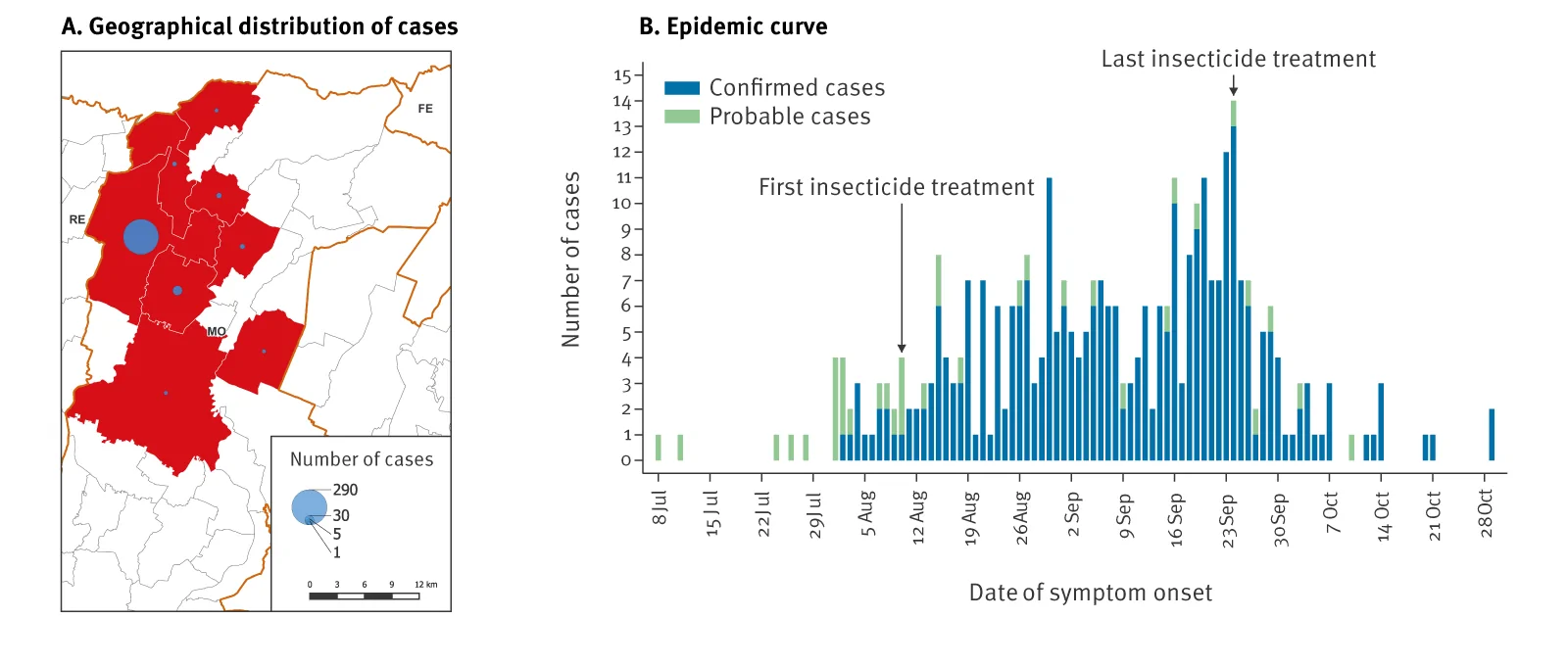
Geographical distribution (A) and epidemic curve (B) of probable (n = 37) and confirmed (n = 306) cases of chikungunya virus infection, by date of symptom onset, Emilia-Romagna region, Italy, August–October 2025

FE: Ferrara; MO: Modena; RE: Reggio nell’ Emilia.

Between 8 August and 31 October 2025, 673 suspected cases were reported to the regional authorities. Chikungunya was diagnosed in 343 (51.0%) of suspected cases: 306 (45.5%) were confirmed and 37 (5.5%) were probable cases. Confirmed and probable cases were residing in eight municipalities of the province of Modena: most in Carpi (n = 294; 85.7%) and Soliera (n = 28; 8.2%), the other cases (n = 21) in Cavezzo, San Prospero, Novi di Modena, Concordia sulla Secchia, Nonantola and Modena (Figure 1A).

As shown in Figure 1B, the earliest symptom onset was on 8 July and the last on 29 October (Figure 1B). Despite the investigations, we could not identify the primary case. The investigation finished on 26 November. The median age of patients was 61.2 years (interquartile range (IQR): 48.0–75.8 years), and ca 70% of cases were older than 50 years. Sex distribution among the cases was almost equal (Table 1). The predominant symptoms included arthralgia (n = 326; 95.0%) and fever (n = 317; 92.4%), followed by asthenia (n = 251; 73.2%), skin rash (n = 219; 63.8%), myalgia (n = 185; 53.9%) and headache (n = 158; 46%). One patient (0.3%) developed meningoencephalitis. Haemorrhagic complications were not observed. Hospitalisation was rare (n = 7; 2%), and no fatalities were reported. No asymptomatic cases were identified. Longitudinal follow-up of patients is ongoing for the occurrence of chronic clinical manifestations.

**Table 1:** Demographic and clinical characteristics of probable (n = 37) and confirmed (n = 306) cases of chikungunya virus infection, Modena province, Emilia-Romagna region, Italy, August–October 2025

Characteristics	Males (n = 166)		Females (n = 177)		Total (n = 343)	
	n	%	n	%	n	%
Age (years)						
0–9	2	1.2	1	0.6	3	0.9
10–19	10	6.0	6	3.4	16	4.7
20–29	9	5.4	7	4.0	16	4.7
30–39	15	9.0	9	5.1	24	7.0
40–49	14	8.4	30	16.9	44	12.8
50–59	30	18.1	29	16.4	59	17.2
60–69	23	13.9	34	19.2	57	16.6
70–79	42	25.3	40	22.6	82	23.9
≥ 80	21	12.7	21	11.9	42	12.2
Median	61.1		61.3		61.2	
Interquartile range	43.5–76.2		48.7–75.3		48.0–75.8	
Clinical symptoms						
Arthralgia	157	94.6	169	95.5	326	95.0
Fever	159	95.8	158	89.3	317	92.4
Asthenia	124	74.7	127	71.8	251	73.2
Skin rash	96	57.8	123	69.5	219	63.8
Myalgia	90	54.2	95	53.7	185	53.9
Headache	80	48.2	78	44.1	158	46.1
Retro-orbital pain	10	6.0	16	9.0	26	7.6
Arthritis	5	3.0	6	3.4	11	3.2
Meningoencephalitis	1	0.6	0	0.0	1	0.3
Hospitalisation						
Yes	2	1.2	5	2.8	7	2.0

### Entomological findings

**Figure 2:**
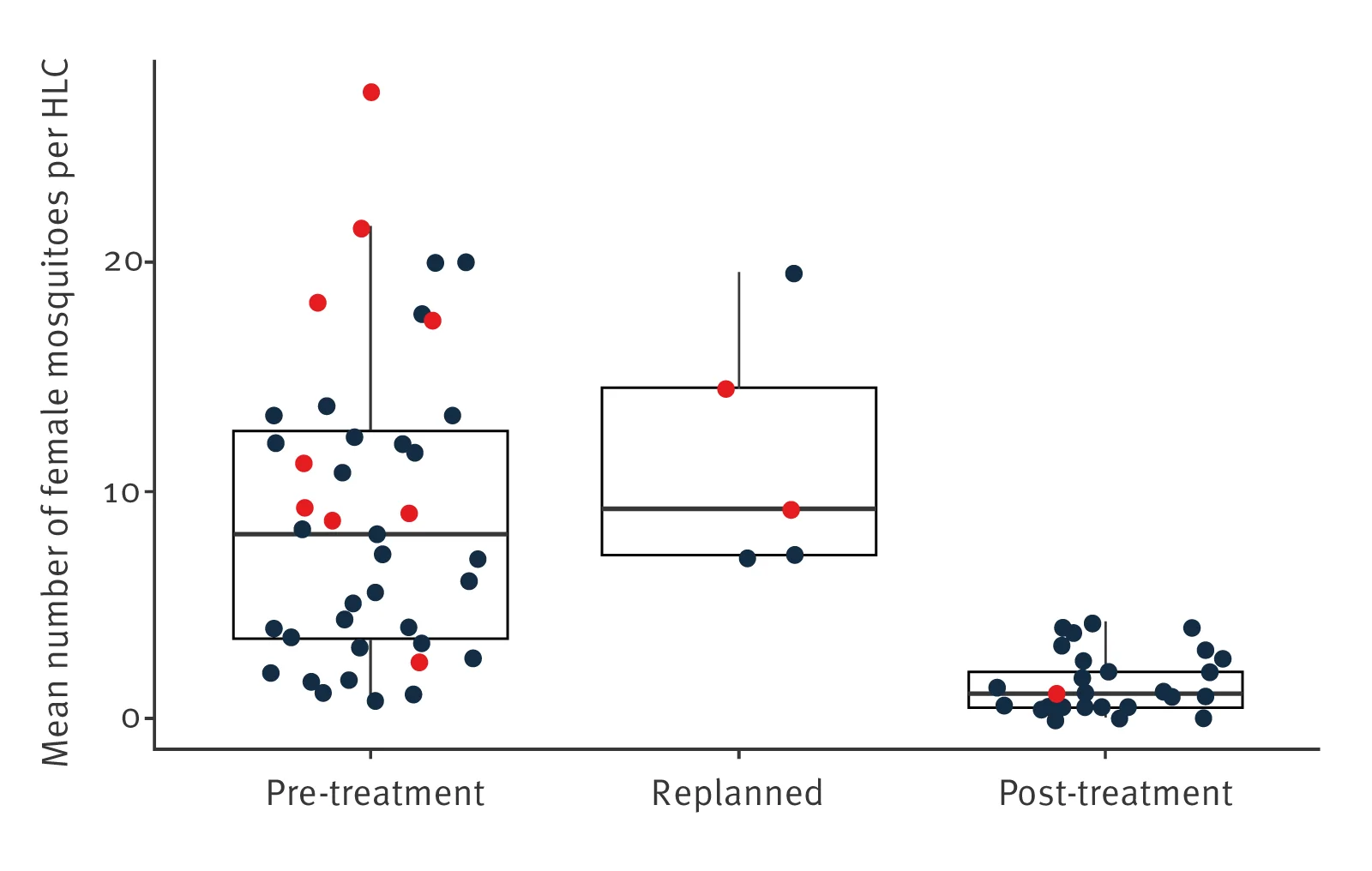
Mean number of collected female mosquitoes per sampling, by treatment, Emilia-Romagna region, Italy, August–October 2025 (n = 2,364)^a^

A total of 2,714 mosquitoes (2,364 females and 350 males) were collected on 73 sampling sessions (pre- and post-treatment). Most mosquito samplings were carried out in the municipality of Carpi (n = 63), with the other samplings (n = 10) in the neighbouring municipalities. The mean number of female mosquitoes collected was 9 ± 7 in pre-treatment and 1 ± 1 in post-treatment samplings (Figure 2), indicating a marked reduction in vector density after the interventions. Female mosquitoes were mixed into 263 pools, and 39 (14.8%) pools tested positive for CHIKV RNA (Table 2). A mean of 13 ± 8 host-seeking females per sampling location was collected in CHIKV-positive pools. Male mosquitoes were tested in 55 pools, with no positive results. Of the 15 mosquito pools collected on 11 August, shortly after the first cases were identified, 13 tested positive for CHIKV, giving a maximum likelihood estimation of the IR of 16.7%. In the subsequent samplings, IR ranged between 10.4% and 0.9% (Table 2). Test-positive pools were predominantly from pre-treatment samplings (31/39). Of the 26 pools from post-treatment samplings, one tested positive for CHIKV RNA; while seven pools were positive in two samplings after replanned treatments (Table 2, Figure 2).

HLC: human landing catch.

^a^ Mosquitoes were tested in 263 pools.

Samplings in which at least one positive pool tested positive are marked in red.

**Table 2:** Mosquito sampling sessions in which at least one pool tested positive for chikungunya virus, Modena province, Emilia-Romagna region, Italy, August–September 2025

Date	Pre- or post-treatment	Municipality	Females (n)	Mean number of females sampled per HLC	Pools (n)	CHIKV detected (n)	MLE IR (%)	Lower limit	Upper limit
11 Aug	Pre	Carpi	150	21	15	13	16.7	9.48	30.92
14 Aug	Post^a^	Carpi	145	15	15	3	2.1	0.57	5.79
17 Aug	Pre	Carpi	90	9	9	2	2.3	0.43	7.73
19 Aug	Post^a^	Carpi	91	9	10	4	5.3	1.77	12.87
22 Aug	Pre	Carpi	70	18	7	5	10.4	4.09	25.08
26 Aug	Pre	Carpi	46	9	5	2	4.9	0.93	16.86
2 Sep	Pre	Carpi	56	11	6	1	1.8	0.11	8.81
5 Sep	Pre	Carpi	43	9	4	1	2.3	0.14	11.47
18 Sep	Pre	Carpi	10	3	1	1	NA		
18 Sep	Pre	Carpi	73	18	8	4	7.0	2.36	17.43
7 Sep	Post	Modena	5	1	1	1	NA		
14 Sep	Pre	Cavezzo	220	28	22	2	0.9	0.17	3.04

CHIKV: chikungunya virus; HLC: human landing catch; IR: infection rate; MLE: maximum likelihood estimation; NA: not applicable.

^a^ The insecticide treatment was replanned because it did not pass the quality control.

### Virological and serological results from case samples

Samples from all suspected cases were tested with molecular and serological assays. The time interval between symptom onset and laboratory diagnosis ranged from 1 to 60 days, with a median of 4 days. Most probable and confirmed cases (n = 268; 78.1%) were diagnosed within 6 days of symptom onset.

**Figure 3:**
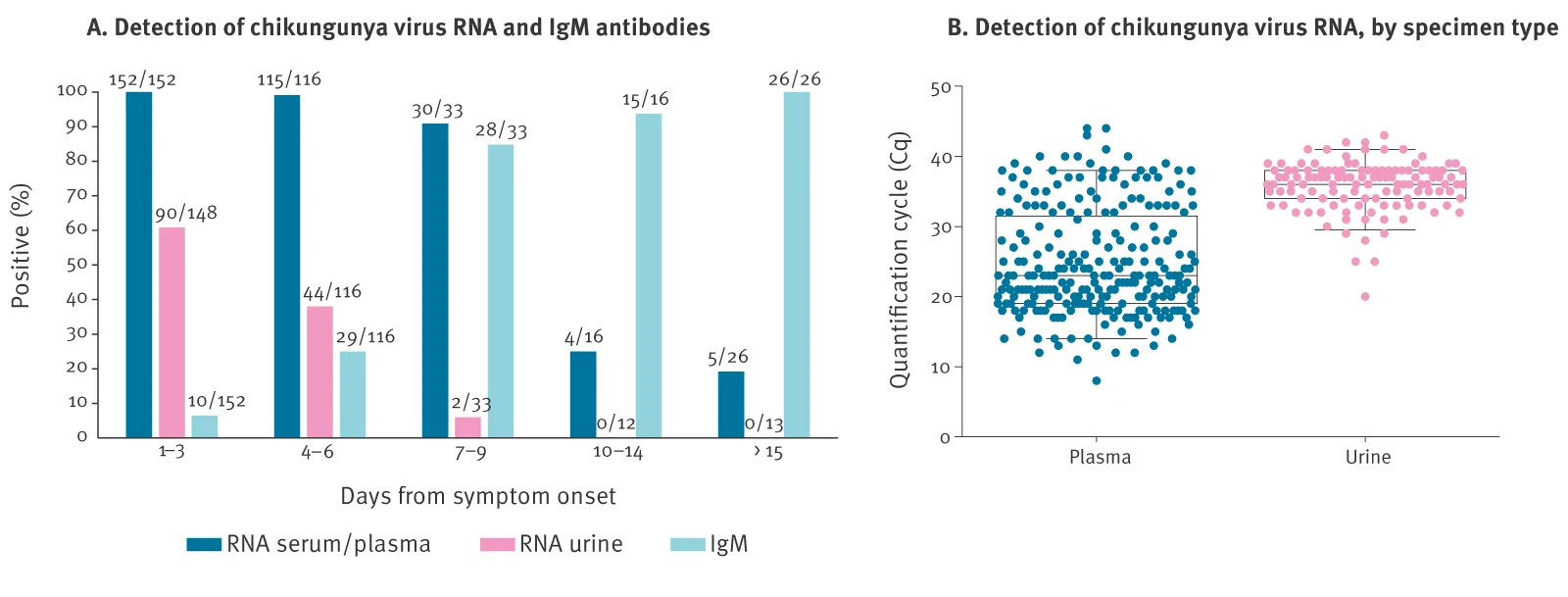
Detection of (A) chikungunya virus RNA and IgM antibodies in probable and confirmed cases of chikungunya infection (n = 343) and (B**)** viral RNA load in blood (n = 343) and urine (n = 322) samples, Modena province, Emilia-Romagna region, Italy, August–September 2025

Chikungunya virus RNA was detected in samples from all confirmed cases. We investigated the kinetics of CHIKV RNA detection in serum or plasma samples (n = 343) and urine samples (n = 322), as well as the detection of IgM antibodies in serum samples (n = 343) (Figure 3A). Overall, CHIKV RNA was detected in 89.2% (306/343) of the tested serum or plasma samples. Over 99% (115/116) of patients tested within 6 days from symptom onset had detectable CHIKV RNA in their serum or plasma samples (Figure 3A). Chikungunya virus RNA was detected in all 152 serum or plasma samples taken 1–3 days from symptom onset, in 30 of 33 samples taken 7–9 days from symptom onset and in 5 of 26 samples taken 15 days from symptom onset (Figure 3A).

In total, CHIKV RNA was detected in 42.2% (136/322) of urine samples during the early viraemic phase (1–3 days from symptom onset: 90/148; 60.8%) and less frequently thereafter (7–9 days after: 2/33; ≥ 10 days after: 0/25). Viral load levels were consistently higher in serum or plasma samples than in urine samples (Figure 3B).

Antibodies (IgM) against CHIKV were detected in 31.5% (108/343) of blood samples from confirmed and probable chikungunya cases. At the beginning of the infection, 1–3 days after symptom onset, 10 (6.5%) of 152 cases had IgM antibodies. In the samples taken later during the infection, IgM antibodies were more often detected (4–6 days after: 29/116 (25%); 7–9 days after: 28/33; 10–14 days after: 15/16; > 15 days: 26/26) (Figure 3A).

### Sequencing and phylogenetic analysis

**Figure 4:**
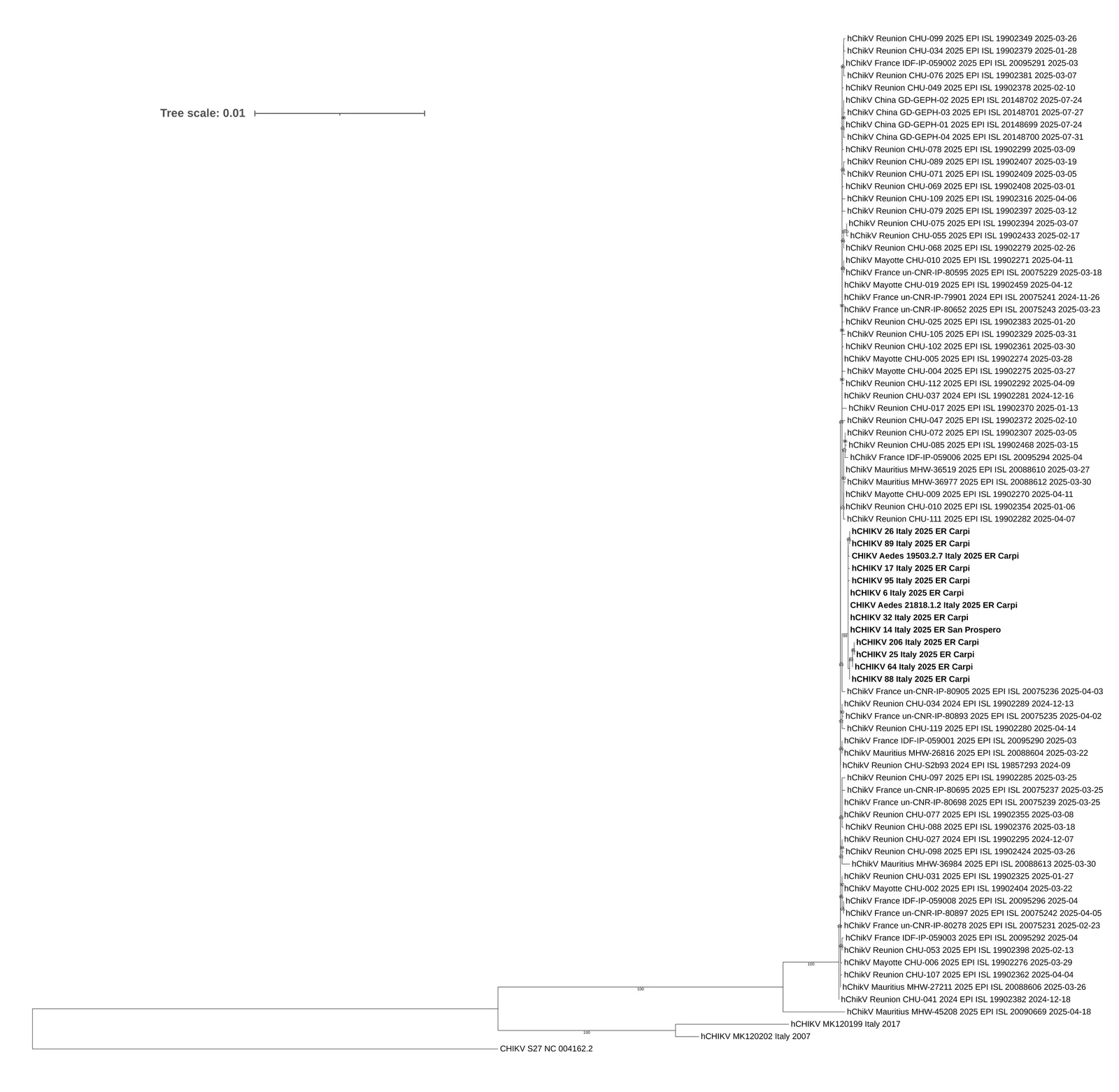
Maximum likelihood phylogeny of full-length genome sequences of chikungunya virus from mosquitoes (n = 2) and autochthonous cases (n = 11), Modena province, Emilia-Romagna region, Italy, 2025 and from Reunion Island, 2024–2025 (n = 67) and from Italy, 2007–2017 (n = 2)

Phylogenetic analysis of 11 sequences from case samples and two from *Ae. albopictus* pools within the Carpi cluster confirmed that the CHIKV strain belonged to the East/Central/South African (ECSA) genotype, lineage 2. The strains exhibited a high degree of genetic similarity and formed a tight cluster within the recently circulating ECSA lineage 2 (ECSA-2). This lineage has been associated with large outbreaks in the Indian Ocean region, such as in Reunion Island in 2024-25, and with subsequent travel-related introductions into Europe [21] (Figure 4). This strain differed from the one circulating in Emilia-Romagna in 2007, which belonged to the ECSA-IOL (Indian Ocean lineage) and carried the E1-A226V mutation [4], and from the 2017 Lazio outbreak strain, also ECSA-IOL, which clustered on a distinct phylogenetic branch and lacked the E1-A226V mutation [6] (Figure 4). The analysis of the viral sequences from the current outbreak revealed the presence of the following critical mutations in all sequenced autochthonous isolates: the mutation E1-A226V, historically known to considerably increase CHIKV infectivity and dissemination efficiency in *Ae. Albopictus*, and the mutations E2-L210Q and E2-I211T, commonly observed in the ECSA-2 lineage, which are believed to act synergistically with E1-A226V, further optimising viral transmission and replication kinetics within *Ae. albopictus* [21]. The phylogenetic analysis was performed to investigate genetic relatedness and clustering patterns; no phylogeographic reconstruction or molecular clock analysis was conducted.

The sequences from the outbreak of this report are marked in bold.

## Outbreak control measures

After the index case was confirmed on 10 August 2025, the population was advised to take measures to avoid mosquito bites and to remove all potential breeding sites from their gardens. Personal protective measures, including the use of insect repellent and mosquito nets, were recommended to prevent onward transmission. Confirmed cases were advised to self-isolate for 7 days from the onset of symptoms which corresponds to the typical viraemic period. Self-isolation was also recommended for household contacts and close contacts identified as suspected cases, until the laboratory testing ruled out the infection. Case resolution was defined by clinical improvement alongside the end of the viraemic phase, lasting approximately 7 days. The CHIKV NAT test was introduced as a preventive blood safety measure in different areas following the spread of the infection.

As stated in the PSCA-ER, the emergency vector control protocol measures were taken within a radius of 100–300 m around the residence of a probable or confirmed case, consisting of: (i) adulticide treatments in public areas in the early morning for three consecutive days, covering streets and green spaces; (ii) door-to-door treatments of permanent mosquito breeding sites (e.g. catch basins) with adulticides and larvicides and the removal of temporary ones (e.g. various artefacts) in the courtyards and gardens of private properties; and (iii) larvicide treatment of road drains. Various authorised commercial pyrethroid-based products (cypermethrin, prallethrin, deltamethrin, tetramethrin and permethrin) were used for adulticide treatments. Silicone-based monomolecular films, *Bacillus thuringiensis israelensis, Lysinibacillus sphaericus* and S-methoprene were used for larvicide treatments. The first insecticide treatment was conducted on 9 August, immediately after the reporting of the suspected index case. After this, the vector control measures continued daily for further 46 days. Following the detection of other cases in the areas of the city where entomological surveillance had revealed high densities of vectors and CHIKV-positive mosquito pools and which had not previously been subject to door-to-door treatment, the crisis unit defined the extension of the area to be targeted by treatment within 48 h. In accordance with PSCA-ER, entomological monitoring continued throughout the outbreak response to assess the quality and efficacy of vector control interventions, by comparing *Ae. albopictus* densities in both public and private areas before and after treatment (Figure 2), thereby providing evidence to guide and adapt vector control strategies. These measures entailed the meticulous monitoring of residual adult and larval mosquitoes within the affected areas. If the control results indicated increased vector densities, additional adulticide treatments were planned and implemented without delay. During the early phases of the outbreak, five treatments had low effectiveness and were immediately replanned (Figure 2). The last treatment was carried out on 24 September. On 25 September, the crisis unit decided to discontinue the adulticide and larvicide treatments as the temperature had decreased and the entomological monitoring showed a sharp decrease in the density and activity of *Ae. albopictus*.

## Discussion

During the summer of 2025, there was an extraordinary increase in locally acquired cases of chikungunya in mainland Europe, with the highest numbers reported in France and in Italy [9-12]. These autochthonous transmission events in mainland Europe occurred alongside the large CHIKV epidemic in Reunion Island and Mayotte from 2024 to 2025 [8], highlighting the intrinsic link between the risk of autochthonous CHIKV transmission in Europe and the intensity of epidemic activity in endemic regions. This increases the probability of the virus being successfully introduced into receptive geographical areas by viraemic travellers.

The 2025 outbreak in Emilia-Romagna represents a large locally transmitted CHIKV event in Italy, following previous outbreaks in the same region in 2007 and in Lazio in 2017 [3-6]. The viral strain responsible for the 2025 Emilia-Romagna outbreak belongs to the ECSA-2 lineage and is genetically related to strains associated with the 2024–25 epidemic in the Indian Ocean region, particularly in Reunion Island [21]. This supports the hypothesis that the primary case was a viraemic traveller, as large outbreaks in endemic regions can act as drivers of importation events into non-endemic areas [22]. The 2025 Emilia-Romagna strain is phylogenetically distinct from the strains of the 2007 and 2017 outbreaks in Italy [4,6], confirming that, so far, the Italian outbreaks have resulted from an independent viral introduction rather than prolonged cryptic local circulation. The tight clustering of the 2025 Emilia-Romagna outbreak sequences within the ECSA-2 lineage, consistent with epidemiological evidence, support the hypothesis of a recent single introduction linked to strains circulating in the Indian Ocean region in 2024-25. A dedicated study employing a Bayesian time-scaled phylogeographic approach will address the precise origin and timing of the virus introduction in Emilia-Romagna region during 2025.

As in the previous outbreaks in France and Italy, the cases were predominantly older adults presenting with arthralgia and fever [3,4,6,7,12]. The higher proportion of older adults may be due to behavioural factors, such as spending more time outdoors during the day, lower adherence to personal protective measures and a higher probability of seeking medical attention upon symptom onset. For detection of CHIKV RNA during the acute phase, serum and plasma samples were superior to urine samples, as reported previously [23,24]. The rapid rise in IgM titres approximately 1 week after symptom onset supports the complementary role of serology in confirming infection when RNA levels are declining. These findings reinforce the value of an integrated molecular and serological diagnostic approach, which is essential for the timely confirmation of cases and prompt initiation of epidemiological and entomological investigations.

The response to the 2025 Emilia-Romagna CHIKV outbreak was based on the integrated framework set out in the national and regional plans for arbovirus surveillance and control [13,14]. This framework included entomological surveillance, vector control, clinical surveillance, contact tracing and increased laboratory capacity. The inclusion of non-endemic arboviruses, such as CHIKV, in the clinical differential diagnosis and laboratory tests was instrumental in identifying the index case and subsequent rapidly activating the public health response. This represents a critical and replicable model for early outbreak detection in settings at risk of importation and local transmission. The rapid identification of cases worked in close synergy with entomological investigations, providing timely data on vector abundance and virus detection in mosquito pools. These data directly guided the prioritisation and targeting of vector control measures, enabling a more focused and efficient deployment of resources in the most affected areas. Post-treatment monitoring indicated a reduction in vector density and identified only one positive mosquito pool, suggesting that vector control interventions, when adequately performed, were effective overall. However, the persistence of infected mosquitoes despite treatment highlights the inherent difficulty of achieving complete viral suppression during an active outbreak. The absence of test-positive male mosquitoes may indicate limited vertical transmission in the vector population, although this observation requires confirmation through larger-scale entomological sampling.

The recent development of vaccines against chikungunya represents a step forward in the prevention of arboviral diseases. However, their implementation in Europe is heterogeneous and subject to national policy decisions. In June 2024, the European Medicines Agency (EMA) authorised the live-attenuated Ixchiq vaccine for individuals aged ≥ 18 years (extended to ≥ 12 years in 2025–26), and in February 2025, the inactivated VLP Vimkunya vaccine was authorised for individuals aged ≥ 12 years [25,26]. The first deployment of Ixchiq occurred in early 2025 in response to the severe outbreak in Reunion Island and Mayotte, prioritising individuals aged ≥ 65 years [27]. In May 2025, EMA restricted the use of Ixchiq for individuals aged ≥ 65 years due to severe adverse events, while maintaining its use for individuals aged < 65 years who are not immunosuppressed. The Ixchiq vaccine is not currently available in Italy due to an ongoing EMA review [25]. The Vimkunya vaccine, approved by the Italian Agency for Medicines (AIFA) in May 2025, has been available since late October 2025. Therefore, it was not commercially available in Italy at the time of the current outbreak. The Vimkunya vaccine is recommended for individuals at increased risk of exposure, such as travellers to endemic areas or populations involved in outbreak settings (age > 65 years, comorbidities). Future outbreak mitigation strategies in Italy should consider proactively integrating of vaccination into national preparedness frameworks, combining immunisation with robust vector surveillance and timely clinical response.

A critical lesson from this outbreak is the time lag between the start of viral circulation, retrospectively traced to the first half of July, and the identification of the index case. This interval enabled *Ae. albopictus* to sustain widespread transmission for an extended period before vector control measures could be implemented. Similar findings were observed during the 2017 outbreak in Italy [5] and the 2025 outbreak in Bergerac, France [12]. Therefore, surveillance systems should be strengthened to reduce this critical interval by increasing awareness of arboviral infections traditionally considered to be geographically remote, and by promoting their early recognition among healthcare professionals and the general population. During the early phases of the outbreak, vector control interventions failed quality controls checks which compromised the effectiveness of the initial response and required immediate replanning. These observations highlight the need to incorporate rigorous quality assurance procedures into vector control protocols, to prevent implementation failures during the acute phase of future outbreaks.

## Conclusions

The large chikungunya outbreak in the Emilia-Romagna region in 2025 highlights the urgent need for sustained and comprehensive preparedness in European areas where *Ae. albopictus* is established. This is particularly important given the projected increase in the likelihood of arbovirus outbreaks in temperate regions. The outbreak further emphasises the importance of integrated surveillance systems encompassing early clinical suspicion, heightened physician awareness, entomological monitoring, timely and effective vector control measures, rigorous epidemiological investigations for case detection and contact tracing, and enhanced laboratory diagnostic capacity.

## Data Availability

Sequences are deposited in GISAID with the following accession numbers: EPI_ISL_20393570, EPI_ISL_20393567, EPI_ISL_20393564, EPI_ISL_20393569, EPI_ISL_20393574, EPI_ISL_20393571, EPI_ISL_20393573, EPI_ISL_20393566, EPI_ISL_20393565, EPI_ISL_20393568, EPI_ISL_20393572; on GenBank with the following accession numbers: PZ196846, PZ196847.
